# In‐situ measurements of tensile forces in the tibialis anterior tendon of the rat in concentric, isometric, and resisted co‐contractions

**DOI:** 10.14814/phy2.13245

**Published:** 2017-04-18

**Authors:** Martin Schmoll, Ewald Unger, Hazel Sutherland, Michael Haller, Manfred Bijak, Hermann Lanmüller, Jonathan C. Jarvis

**Affiliations:** ^1^ Center for Medical Physics and Biomedical EngineeringMedical University of ViennaViennaAustria; ^2^ School of Sport and Exercise ScienceLiverpool John Moores UniversityLiverpoolUnited Kingdom

**Keywords:** Antagonistic co‐contraction, in‐line force‐measurement, rat tibialis anterior

## Abstract

Tensile‐force transmitted by the tibialis anterior (TA) tendon of 11 anesthetized adult male Wistar rats (body‐mass: 360.6 ± 66.3 g) was measured in‐situ within the intact biomechanical system of the hind‐limb using a novel miniature in‐line load‐cell. The aim was to demonstrate the dependence of the loading‐profile experienced by the muscle, on stimulation‐frequency and the resistance to shortening in a group of control‐animals. Data from these acute‐experiments shows the type of loading achievable by means of implantable electrical stimulators activating agonists or agonist/antagonist groups of muscles during programmed resistance‐training in freely moving healthy subjects. Force‐responses to electrical stimulation of the common peroneal nerve for single pulses and short bursts were measured in unloaded and isometric contractions. A less time‐consuming approach to measure the force‐frequency relationship was investigated by applying single bursts containing a series of escalating stimulus‐frequencies. We also measured the range of loading attainable by programmed co‐contraction of the TA‐muscle with the plantar‐flexor muscles for various combinations of stimulation‐frequencies. The maximal average peak‐force of single twitches was 179% higher for isometric than for unloaded twitches. Average maximal isometric tetanic‐force per gramme muscle‐mass was 16.5 ± 3.0 N g^−1^, which agrees well with other studies. The standard and time‐saving approaches to measure the force‐frequency relationship gave similar results. Plantar‐flexor co‐activation produced greatly increased tension in the TA‐tendon, similar to isometric contractions. Our results suggest that unloaded contractions may not be adequate for studies of resistance‐training. Plantar‐flexor co‐contractions produced considerably higher force‐levels that may be better suited to investigate the physiology and cell‐biology of resistance‐training in rodents.

## Introduction

There are 24 muscles in the mammalian hind limb that act together to produce coordinated patterns of movement (Charles et al. 2016). The force produced by individual muscles is determined by the number of its constituent motor units that are activated, by the frequency of the activating impulses, and by the resistance against which it generates force, according to the well‐established relationship between force and velocity (Hill 1938; Ranatunga 1984; Jarvis 1993). The maximum intrinsic force is generated when all motor units are recruited at a frequency for which the responses to the activating pulses summate into a smooth tetanic contraction (Simard et al. 1982), and when the muscle is unable to shorten because the resistance to shortening is greater than the maximum force that the muscle can produce. If the muscle is able to shorten, then the force is reduced according to the approximately parabolic force‐length relationship (Rassier et al. 1999). Even greater forces can be experienced by the muscle if it is stretched during activation by an external force during eccentric contractions.

In normal movement, muscles are required not only to produce rotation of the limb segments around the joint centers, but also to stabilize those flexible couplings. Consequently, normal movement involves co‐contraction of antagonist muscles, so that these muscles resist one another. In this situation the resultant force, measurable from outside the limb, cannot easily be deconstructed into the component forces of the contributing antagonistic sets of muscles. Assessing muscular activity within the intact biomechanical system remains a challenging task.

Although the recording of electromyographic (EMG) activity is a valuable and easily accessible technique to estimate muscular activity, it is difficult to give reliable statements about how the measured signal is correlated to the absolute force values produced by the observed muscle (Hof 1997). As EMG is usually measured using surface or intra‐muscular needle electrodes, they may only record a part of the activity of a particular muscle. Also, co‐activation of muscles close to the targeted one, may cause artefacts confounding such measurements. Measuring the muscular force provides a more complete picture of the activity of a certain muscle.

Several different approaches have been used in the past to assess force in the hind limb muscles of small laboratory animals. These muscles are widely used models to investigate muscular behavior, as they are large enough to manipulate and their nerves are accessible. Besides describing contractile properties of a muscle (Jarvis and Salmons 1991; Biewener and Baudinette 1995; Biewener 1998; Daley and Biewener 2003; Shin et al. 2008), measured force is also used as an essential parameter to quantify the degree of nerve regeneration after injury (Spyropoulos et al. 1994). Further it is possible to gain objective data to assess the influence of pathologic conditions on the ability to generate force (Edwards 1980; Gibson et al. 1993). Other studies have measured force responses elicited by various electrical stimulation parameters to investigate strategies for selective recruitment of fast or slow motor units (Gorman and Mortimer 1983; Grill and Mortimer 1996; van Bolhuis et al. 2001). The latter could lead to electrical stimulation protocols that activate the muscle more nearly according to the recruitment strategies intrinsic in the native neuromuscular system. Also the investigation of training protocols to elicit adaptation of muscle properties is a well‐established field of research (Gonyea and Bonde‐Petersen 1978; Duncan et al. 1998; Sutherland et al. 2003; Vickerton et al. 2014), but many questions concerning the relationship between the pattern of activity and the rate and extent of transformation remain.

Functional electrical stimulation of the motor nerves allows for close control of the pattern of additional activity. This serves as a powerful tool for eliciting well‐defined contractions with a large range of applications (Gonyea and Bonde‐Petersen 1978; Edwards 1980; Gorman and Mortimer 1983; Jarvis and Salmons 1991; Gibson et al. 1993; Spyropoulos et al. 1994; Grill and Mortimer 1996; Marsh et al. 1998; van Bolhuis et al. 2001; Sutherland et al. 2003; Shin et al. 2008; Grasa et al. 2014; Vickerton et al. 2014). Salmons and Vrbová (1967) introduced a model of neuromuscular adaptation by means of implantable electrical nerve stimulators. The early devices produced a constant stream of impulses. Such continuous patterns delivered at the typical discharge frequency of “slow” motor neurons induced changes in the muscles that demonstrated the remarkable ability of adult skeletal muscle fibers to transform reversibly across the spectrum of fiber type from fast, glycolytic fibers to slow oxidative fibers.

New developments in miniature integrated electronic circuits mean that implanted devices can now deliver complex daily patterns of nerve stimulation with full control of frequency, amplitude, and temporal pattern. Most previous studies (Jarvis 1993; Sutherland et al. 1998) of muscle activation with implantable stimulators have used stimulation of the dorsi‐flexors of the foot via the common peroneal nerve. In a normal sedentary position, then, the muscle is working only against the load represented by the mass of the foot itself and there is no direct way to measure the force generated by the muscle in this situation.

To measure the force generating capacity of an individual muscle it is necessary to arrange for that force to act on a sensor. Usually the tendon of origin is stabilized by a clamp and the tendon of insertion is cut and connected to a fixed transducer for the measurement of isometric force (Duncan et al. 1998; Marsh et al. 1998; van Bolhuis et al. 2001; Sutherland et al. 2003; Shin et al. 2008), or to a lever system that can provide controlled loads or velocities of movement during activation (Faulkner et al. 1989; Jarvis and Salmons 1991). However, because the tendon of insertion is cut, the link between the muscle of interest and the other muscles that act on the same biomechanical system is broken. A newly developed system allows us to measure the force in the tibialis anterior (TA) tendon in the anaesthetized rat without breaking that link. This enables the measurement of forces generated by the TA muscle during contractions in which the muscle only lifts the foot, during contractions in which the foot is held at a fixed angle to the tibia so that the muscle acts isometrically, and during contractions in which the plantar‐flexors are also activated and therefore provide a resisting force to the contraction of the tibialis anterior muscle.

On the basis of such measurements it will be possible to design programmes of daily activity using implantable electrical stimulators in which the TA muscle is activated to contract against different loads without needing to provide any external resistance. This will be a useful model with which to investigate the relationships between load, and the daily pattern of activity in exercises designed to induce hypertrophy of the muscle.

The primary goal of this study was to estimate the type of loading on the TA muscle that might be achieved with implantable electrical nerve stimulators during programmed resistance training in the freely moving rat. The recruitment characteristics in response to single stimuli, and the relation between stimulating frequency and developed force was assessed for the TA muscle using a novel in‐line load‐cell. These measurements were performed in acute experiments in anesthetized rats for the unloaded (UNL) case in which the foot was unrestrained and for the isometric (ISO) case in which the ankle joint was held at a constant position.

High muscular tension has proven to be a well‐known anabolic factor within the scope of resistance training. It is possible that simple unloaded contractions do not induce a sufficiently high force signal to elicit the change in the balance between protein synthesis and degradation that is required to induce hypertrophy. Higher loading might be expected with co‐contraction of the antagonistic muscles, transferring additional tension via the ankle joint. To quantify these forces additional measurements were performed in which both the dorsiflexor and plantarflexor muscle groups acting on the foot were activated.

## Methods

### Animals and surgical procedure

All experiments were conducted in strict accordance with the Animals (Scientific Procedures) Act of 1986, governing animal experiments in the UK. The procedures performed in this study were approved by the Home Office (PPL 40/3280), and were in compliance with the Physiological Society's policy on animal experimentation (Drummond 2009). The 12 adult male Wistar rats which were used in terminal experiments were bred within our local animal unit. Animals were group‐housed maintaining an alternating 12 h light 12 h dark cycle, providing food and water ad libitum.

In 11 rats (age 109 ± 49 days) the forces in the TA tendon during stimulation of the common peroneal nerve were measured. One further experiment demonstrated the effect of co‐contraction (CC) of the plantar‐flexors by simultaneous stimulation of the common peroneal and tibial nerves.

All animals were anaesthetized using a gaseous mixture of isoflurane and O_2_. An initial concentration of 4% was used for induction and was adjusted individually to levels of 1–2% to maintain adequate anesthesia. Buprenorphine (Temgesic, Indivior, Slough, UK) at 0.05 mg kg^−1^ body mass, was administered intramuscularly for analgesia.

**Figure 1:**
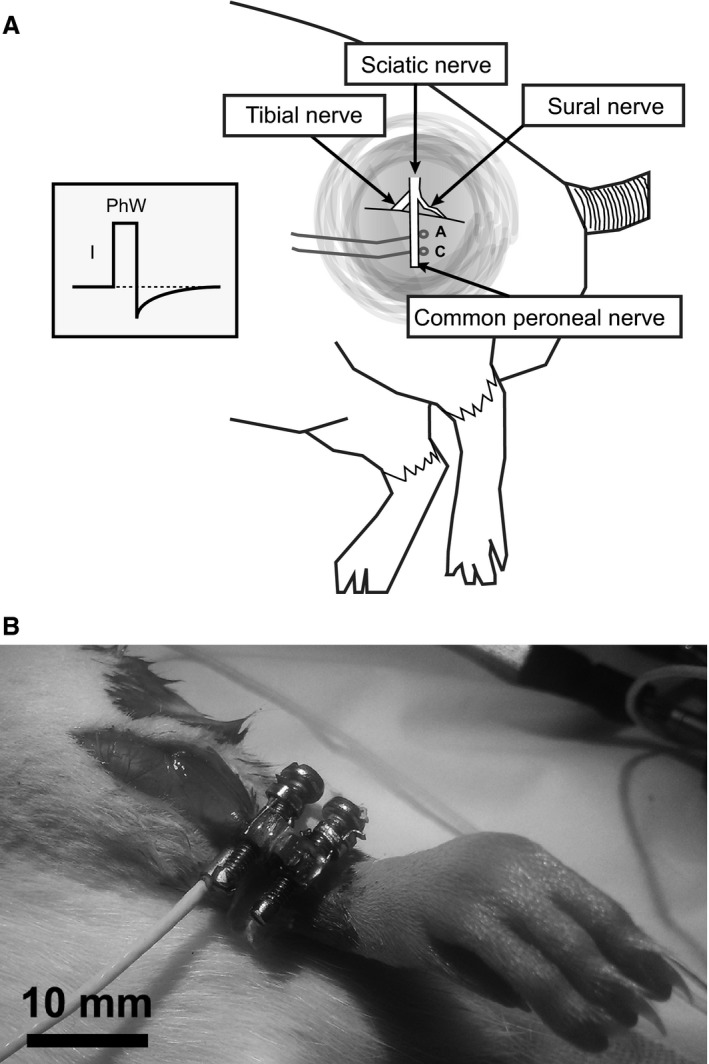
(A) Positioning of the loop‐electrodes used for electrical stimulation of the common peroneal nerve along with a representation of the current‐controlled waveform of a typical stimulation pulse with a certain amplitude I and phase‐width (PhW). (B) Typical measurement situation with load‐cell attached in‐line with the tendon of the tibialis anterior muscle.

Two loop electrodes, formed from PVC‐insulated stainless steel wires (Cooner Sales Company, Chatsworth, California, U.S.A.), were placed under the common peroneal nerve with the more distal electrode as cathode (Fig. 1A). For the experiment in which co‐contraction was tested, an additional pair of equivalent electrodes was placed under the tibial nerve. The incision was closed in layers after electrode placement to maintain the nerves in their natural environment.

The tibialis anterior muscle was accessed through a longitudinal skin incision on the anterior part of the lower hind limb. Once the TA was clearly visible, the incision was extended further distal being guided by the tibialis tendon. In order to have a longer tendinous part freely accessible, the retinaculum was transected, taking care to preserve adjacent blood‐vessels.

The cleaned tendon was inserted into a newly developed miniature load‐cell (Center for Medical Physics and Biomedical Engineering, Medical University Vienna, Austria) and clamped using two stainless steel screws as shown in Figure 1B. The load‐cell consisted of a CNC‐manufactured stainless steel core measuring 3 × 3 × 7 mm and was designed to measure tensile forces up to 20 N by using a temperature compensated half‐bridge consisting of four strain gauges (1‐LY11‐0.3/120, Hottinger Baldwin Messtechnik GmbH, Darmstadt, Germany). In order to cause the minimum possible disturbance to the biomechanical system, the overall mass of the device was only ~0.5 g. A protective element was shaped by flattening and bending a 21 G needle around the screws, so that the tendon experienced a lateral clamping force rather than a torsional stress when tightening the screw. This is important because torsional stress tends to cut the fibrous elements of the tendon. By this means the tendon was clamped into the load‐cell without any change in the distance between the muscle and its insertion. The tendon between the two clamping screws was then transected through a small window in the device. This part of the procedure requires the use of optical magnification to be performed safely and completely. Once the tendon was cut, the whole muscle force was transmitted through the transducer itself and could thus be continually monitored, allowing for direct quantitative tensile force measurements. A drying‐process of 20–30 min was allowed to stiffen the tendon at the clamping points. We found this to be necessary to avoid damage to the tendon when applying repeated maximal contraction forces. Inevitably this will have changed the elastic properties in this small area of the tendon.

The animals were killed by cervical dislocation without recovery from anesthesia. TA muscles from the left and right side were harvested and weighed immediately.

### Experimental set up

#### Stimulation equipment

Rectangular monophasic constant current pulses followed by an exponential charge balancing reverse current (see Fig. 1A), were generated with a MiniVStim pulse generator (PG) described in (Bijak et al. 2016). The PG was a freely programmable device, able to deliver complex electrical stimulation patterns in a repeated and autonomous fashion. The associated MiniVStim app, installed on a standard Android powered tablet computer (Xperia Tablet Z, Sony Corporation, Tokyo, Japan), enabled the design and storage of all stimulation patterns that were required in this study. The tablet computer maintained an active Bluetooth connection to a programming device to transfer the predefined stimulation patterns to the pulse generator. Communication between programming device and pulse generator was achieved via a bidirectional radio‐frequency link. Once a pattern was successfully transferred to the pulse generator, execution started automatically.

#### Data recording

A PowerLab 16/35 (ADInstruments Inc., Colorado Springs, USA) device was used to power the load‐cell and measure the force signal at 10 kS/s. Stimulation voltage was recorded at a sample rate of 100 kS/s along with the force signal. ADInstruments LabChart 7 Pro installed on a standard personal computer (MSI GS60 2PE Ghost Pro, Micro‐Star International, Zhonghe District, Taiwan) was used to record, store, pre‐process and export the retrieved data.

### Stimulation protocol

#### Recruitment curves

Single pulses with a phase‐width of 258 *μ*sec were delivered every 3 sec while the stimulation amplitude was increased every other pulse by 0.1 mA, to cover all amplitude levels from 0.1 to 2.0 mA. The mean force response elicited by two consecutive pulses with the same amplitude was used to create a recruitment curve (this is, the force response in relation to stimulation amplitude). In a first step, measurements were performed with the foot free to move, allowing unloaded contractions of the TA muscle. For isometric contractions, the foot was held at 90° to the tibia.

#### Force‐frequency curves

The relationship between developed force of the fully recruited muscle, and stimulation frequency was assessed with short bursts (300 ms) of supramaximal stimulating pulses (2 mA, 258 *μ*sec) at 10, 20, 40, 60, 80 and 100 Hz. To avoid progressive muscular fatigue and to allow for complete recovery, the time between bursts was set to 30 sec. A more time‐efficient approach, similar to the techniques used by other research groups (Edwards 1980; Gibson et al. 1993), is to embed all different frequencies into a single frequency‐ramped burst. This burst was constructed as follows: 2 sec stimulation at 1 Hz, 600 msec at 5 Hz, 500 msec at 10 Hz, 65 msec break (due to technical limitations), 200 msec at 20 Hz, 100 msec at 40 Hz, 100 msec at 60 Hz, 100 msec at 80 Hz and 100 msec at 100 Hz. Both approaches were recorded for UNL and ISO contractions.

#### Co‐Contraction

To investigate the possibility of quantifying the force generated by the tibialis anterior muscle during co‐contraction of the plantar flexor muscles, a second MiniVStim pulse generator with an externally gate‐able output‐stage was used. The primary PG delivered a series of short (500 msec) supra‐maximal (2 mA) bursts at frequencies of 20, 40, 60, 80 or 100 Hz to the common peroneal nerve, while simultaneously activating the output‐stage of the other PG connected to the electrodes placed under the tibial nerve. The second PG allowed for synchronized activation of the plantar‐flexors, which were also stimulated supra‐maximally (2 mA) at frequencies of 20, 40, 60 or 80 Hz. A period of 30 sec rest was introduced between contractions to avoid progressive fatigue. To register even small changes in muscle performance over time, a supra‐maximal (2 mA) control‐burst (duration 200 msec, frequency 100 Hz) was applied to the common peroneal nerve with the foot held at the 90° ankle position, prior to each test‐series to act as a standard contraction and internal control.

### Data analysis and statistics

LabChart was used to mark the beginning of each twitch and burst. This annotated data was exported for further processing with Matlab R2010a (MathWorks, Natick, Massachusetts, United States). A semi‐automatic script was used to evaluate the force produced by the muscle. The manually selected baseline (passive force) was subtracted from the total force‐signal. The maximum developed force F_peak_ (measured peak force minus passive force) was determined for every single response. Results were stored and further processed using Microsoft Excel (Microsoft Corporation, Washington, United States).

One‐way ANOVA with a selected significance level of *α *= 0.05 was used to test statistical differences between groups.

#### Alignment of recruitment curves

The lowest stimulation amplitude that was able to elicit a response with F_peak_ > 0.1 N was considered to be the threshold amplitude, designating the beginning of each individual recruitment curve. As the threshold amplitude is mainly influenced by the position of the stimulating electrodes, it was necessary to align the individual recruitment curves on the amplitude axis, according to their threshold amplitude. Amplitude was then plotted as amplitude‐above‐threshold. These values were also used to calculate mean amplitude levels for the combined recruitment curves.

#### Co‐contraction

F_peak_ was determined for each burst applied. Control‐bursts were considered to produce contractions reflecting the current isometric force maximum of the TA muscle. The control‐burst prior to each test‐series was used to normalize the test‐bursts to take account of any progressive change in the muscle performance.

## Results

### Animals

**Figure 2:**
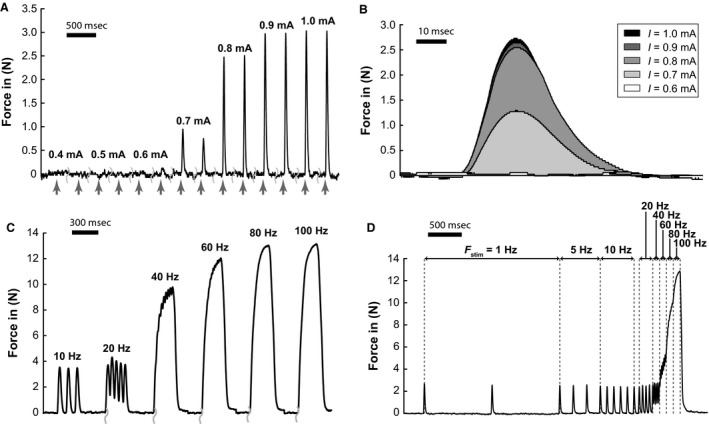
Force recordings obtained with a sample rate of 10 kS/s, taken from representative experiments during isometric contractions. (A) Sample data obtained during the recording of a recruitment curve. Stimulation pulses are marked with arrows. Note: In order to illustrate the recruitment behavior more clearly, the time between consecutive pulses (3 sec) was removed. (B) Sample recordings of single twitches at different amplitudes of stimulation. (C) Sample data for a series of bursts with varying stimulation frequency. Note: In order to illustrate the force frequency relationship more clearly, the time between consecutive bursts (30s) was removed. (D) Recording of a single burst with stimulation frequency (F_stim_) ramped from 1 to 100 Hz.

Muscle mass and force data for the 11 rats in which only the common peroneal nerve was stimulated is presented in Table 1. The rats had an average body mass of 360 ± 66 g. The TA muscles whose mechanical properties had been measured, weighed 695 ± 131 mg. There was no significant difference in the mean weights of stimulated (right) and unstimulated (left) TA (*P* = 0.965). The measurements were achieved, therefore, without any significant drying or edema that would have decreased or increased the wet weight on the experimental side. Representative force traces for the different experiments are illustrated in Figure 2.

**Table 1:** Overview of weights and corresponding peak forces of all animals studied

Animal #	Age (days)	Body weight (g)	LTA (mg)	RTA (mg)	F_peak_ twitch UNL (N)	F_peak_ twitch ISO (N)	F_peak_ tetanic UNL (N)	F_peak_ tetanic ISO (N)	F_peak_/muscle‐weight (N/g)
1	211	457	734	774*	1.4	3.4	5.8	11.9	15.3
2	183	432	858*	840	1.2	3.6	6.0	13.8	16.1
3	93	395	846*	852	1.2	4.6	5.1	11.9	14.1
4	147	469	903*	869	1.3	4.0	9.8	16.5	18.3
5	64	285	533*	548	0.9	1.8	4.5	8.6	16.2
6	65	300	571*	499	0.8	2.4	4.0	7.8	13.7
7	91	304	586*	567	0.9	1.6	5.8	10.6	18.1
8	92	346	624*	617	0.5	1.5	3.5	6.9	11.1
9	92	320	661*	641	1.0	2.6	9.9	13.9	21.0
10	83	338	576*	600	0.8	2.9	5.4	11.9	20.6
11	83	320	723*	780	1.3	3.2	6.3	12.2	16.9
Mean	109	361	692	702	1.0	2.9	6.0	11.5	16.5
SD	49	66	130	137	0.3	1.0	2.1	2.8	3.0

The ‘*’ symbol indicates the investigated side.

### Recruitment curves

**Figure 3:**
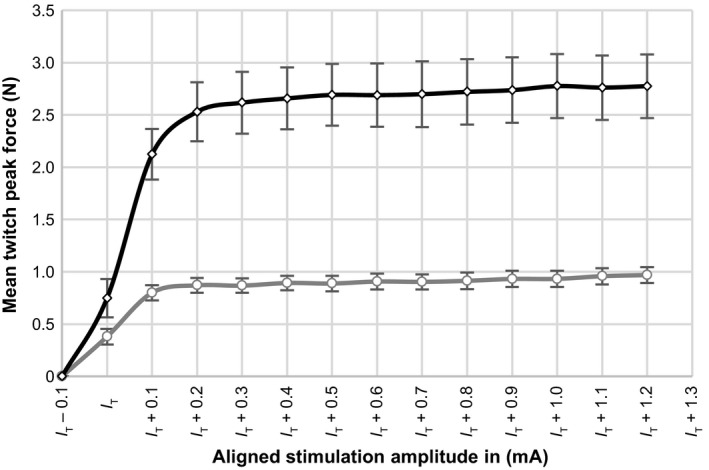
Averaged recruitment curves of the TA muscle (*n* = 11) obtained by single twitches for unloaded (grey) and isometric (black) conditions. Individual recruitment curves were aligned, according to their threshold amplitude (I_T_), before calculating the mean of each amplitude step above threshold. Data presented as mean twitch peak force ± SEM. Mean I_T_ was 0.48 ± 0.16 mA for unloaded and 0.45 ± 0.17 mA for isometric contractions.

Recruitment curves shown in Figure 3 are presented as means with the standard error of the means (SEM) for all tested amplitude levels. It was necessary to align the recruitment curves so there are fewer data‐points for animals that had higher threshold amplitudes. Mean values were calculated for data‐points obtained in all animals. The average threshold amplitude was 0.48 ± 0.16 mA for unloaded and 0.45 ± 0.17 mA for isometric twitches (*P* = 0.7). The maximum twitch force was significantly higher (279%, *P* < 0.01) in the isometric than in the unloaded case.

### Force‐frequency curves

#### Multi‐burst approach

**Figure 4:**
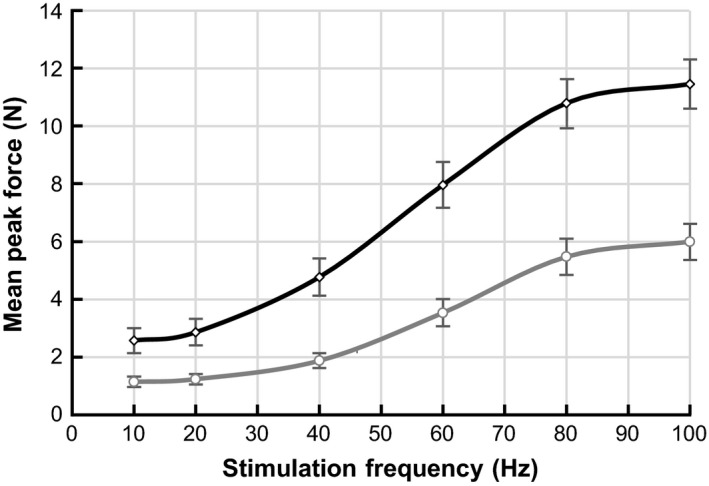
Averaged force‐frequency responses of the TA muscle (*n* = 11) obtained by short bursts at different frequencies for unloaded (gray) and isometric (black) conditions. Data presented as mean peak force ± SEM.

Short supra‐maximal bursts of different frequencies (10, 20, 40, 60, 80 or 100 Hz, separated by 30 sec rest) were applied while the force was monitored. Figure 4 demonstrates the force‐frequency relationship as mean and SEM of the maximal developed force, collected from all 11 rats. Isometric contractions showed a significantly (*P* < 0.01) higher force development for all tested frequencies. The mean peak developed force for unloaded contractions was 52% of the mean peak force developed in isometric contractions.

#### Single‐burst approach

**Figure 5:**
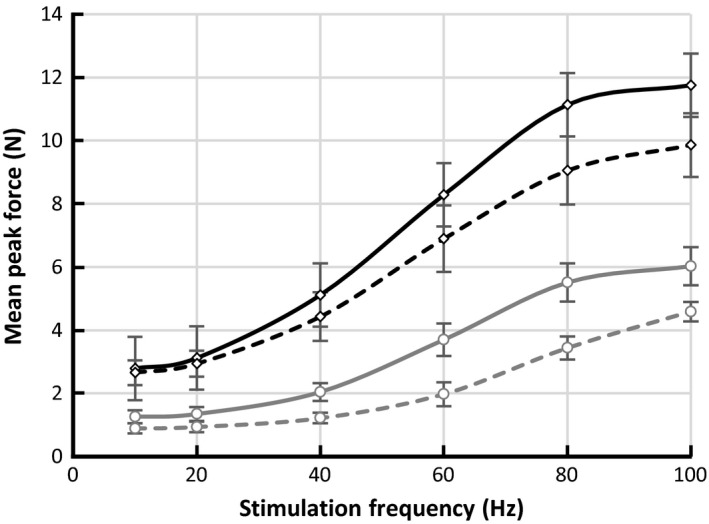
Averaged force‐frequency curves of the TA muscle (*n* = 8) using multiple (solid line) and a single frequency‐ramped (dashed line) bursts. Data is presented for unloaded (gray) and isometric (black) contractions. Data presented as mean peak force ± SEM.

Complete results using frequency‐ramped bursts were obtained from 8 out of the 11 animals, due to some failures of the interface between the tendon and the integral clamp of the load‐cell at higher forces. Figure 5 illustrates the differences between the standard multi‐burst (solid lines) and the alternative frequency‐ramped single‐burst (dashed lines) approach, for unloaded and isometric contractions. The single‐burst approach showed generally lower peak forces compared to the use of multiple bursts but a very similar relationship between the relative forces produced at each frequency. The technique thus gives a rapid estimate of the force‐frequency relationship within a few seconds.

#### Co‐contraction

**Figure 6:**
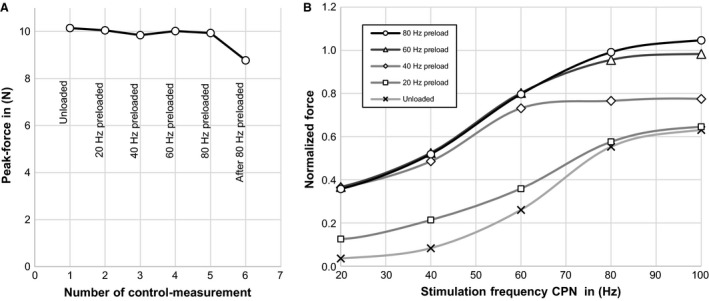
A series of short (500 msec) supra‐maximal (2 mA) bursts of different frequencies (20, 40, 60, 80 and 100 Hz) was delivered to the common peroneal nerve while simultaneously supra‐maximally (2 mA) stimulating the tibial nerve at selected frequencies (20, 40, 60 and 80 Hz). One series was recorded without additional tibial nerve stimulation. (A) Peak‐Forces of short (200 msec) supra‐maximal (2 mA) control‐bursts at a stimulation frequency of 100 Hz applied to the common peroneal nerve while fixing the ankle joint. Control bursts were applied before and after each test‐series. (B) Peak‐forces normalized to their corresponding isometric control‐burst, for various stimulation frequency combinations. Stimulation frequency for the tibialis anterior muscle (common peroneal nerve) is shown on the *x*‐axis, stimulation frequency for the plantar‐flexors (tibial nerve) is shown in the legend (as preload).

Evaluation of the control‐bursts (Fig. 6A) revealed similar values of F_peak_ before and after each experimental series, for all tested preloads except when the antagonistic plantar‐flexors were stimulated at 80 Hz. In this case a decline of 11.7% was observed and we conclude that this combination of stimulation parameters produced a degree of eccentric (lengthening) contractions of the TA muscle that are known to produce an acute reduction in force (Faulkner et al. 1989).

The simultaneous activation of the plantar‐flexor muscles showed higher forces generated in the tibialis anterior tendon (Fig. 6B) compared to the unloaded case. Higher frequencies led to increased tension transmitted by the TA tendon. The highest increases were observed when stimulating the tibial nerve with 80 Hz, and in this case the force measured in the tibialis anterior tendon was similar to the force measured during a control (isometric) contraction, and approximately 60% higher than in the unloaded contractions. These data show that it is possible to provide a graded resistance to the tibialis muscle by means of careful programming of activation in the agonist and antagonist nerves, in the intact biomechanical system of the rat hind limb.

## Discussion

Tensile forces transmitted by the tibialis anterior tendon of rats, either generated exclusively by the TA muscle or in combination with co‐contracting plantar flexors additionally loading the tendon via the ankle joint, were successfully measured in‐situ using a novel miniature in‐line load‐cell. Although the load‐cell was small enough to be attached to the rat′s TA tendon, it was necessary to transect the retinaculum in order to make a longer part of the tendon freely available. This causes a slightly different pathway of force transmission to the dorsal surface of the foot but did not interfere with the full range of motion in the ankle joint. It is therefore a technical limitation of the load‐cell and a stimulus for even greater miniaturization but we believe the data obtained are novel and instructive in understanding the forces acting around the ankle joint in the rat.

The results showed that the load‐cell gave reproducible results over a period of several months in 11 separate experiments. It was possible to measure forces up to 16.5 N (that is the gravitational force acting on a 1.68 kg mass) at a resolution high enough to quantify single twitches.

Complete activation of the TA muscle was achieved using electrical stimulation of the common peroneal nerve. In‐situ measurements of the force transmitted by the tibialis anterior tendon have not been performed previously and provide a clear guide to the type of loading that can be achieved during programmed stimulation in the intact biomechanical system with implantable electrical stimulators.

Values for the maximal isometric tetanic force measured with our device are in agreement with earlier published results using fixed force transducers. When normalizing the maximal isometric force to the wet weight of the stimulated muscle, our results of 16.5 ± 3.0 N g^−1^ are well within the range reported in other studies, for example, 15.9 N g^−1^ (Grasa et al. 2014) (standard deviation not reported), 15.1 ± 1.6 – 16.3 ± 1.0 N g^−1^ (Marsh et al. 1998) and 17.2 N g^−1^ (Shin et al. 2008) (standard deviation not reported), for adult rats. The range of reported values could be related to different muscle lengths, rat strains and the age of the individual animals used.

The use of a single frequency ramped burst might be a time efficient alternative to characterize the force‐frequency relationship of a muscle, within seconds instead of minutes. Although absolute peak forces were slightly lower, the novel approach was still able to reflect a force‐frequency relationship that was comparable to the standard technique. The duration of each frequency step was shorter in the frequency‐ramped burst which gave the muscle less time to develop force. Nevertheless, this was a necessary compromise between time at each frequency point and time required for the compound contraction in order to avoid fatigue within the burst.

The ability to measure the force of a muscle while still acting in its natural biomechanical environment, provides useful additional information, compared to measurements in which the muscle is removed from its original attachments. This is of particular interest when investigating the adaptive muscle‐response to changes in loading (typically over a period of a few weeks) (Gonyea and Bonde‐Petersen 1978; Duncan et al. 1998; Sutherland et al. 2003) or in studies examining of the response of bone to the stresses imposed by muscular contractions (Vickerton et al. 2014). When aiming to use resistance training to achieve muscular adaptation, such as hypertrophy, a general recommendation to use a load corresponding to 70–85% of the one‐repetition maximum load (American College of Sports Medicine, 2009) is commonly used.

In order to design a rational training regime it is therefore important to know about the forces occurring within this system, to select a certain training stimulus that is challenging enough to trigger muscular adaptations without causing irreversible damage to the muscle.

The results of this study show that during contractions in which the TA muscle acts on the unrestrained foot the generated peak forces are approximately half of the maximum isometric tension. Such relatively unloaded contractions, even at high frequencies of stimulation, may not be appropriate for studies of resistance training. A resisting force generated by co‐contracting antagonistic muscles and transmitted through the tendon across the ankle joint could potentially be used to train the tibialis anterior with a range of loading protocols.

The preliminary measurements performed on the CC rat indicate that the additional loading can reach values up to those observed during isometric contractions when fixing the ankle joint. Varying the stimulation frequency delivered to the tibial nerve allows control of the amount of additional loading. Finer adjustment might be achieved by modulating the stimulation amplitude as well. These preliminary measurements support the principle that antagonistic co‐contraction might serve as means to provide high loading of the TA muscle.

## Conclusions

Tensile forces transmitted by the TA‐tendon of rats were successfully measured in‐situ while maintaining the muscle's anatomical position, using a novel miniature in‐line load‐cell. These measurements provide a clear guide to the type of loading that can be achieved in the intact biomechanical system using implantable electrical stimulators. Our results suggest that unloaded contractions, even at high frequencies of stimulation, may not be adequate for studies of resistance training. Simultaneous activation of the antagonistic plantar flexor muscles produced considerably higher force‐levels that may be better suited for electrically‐induced resistance training of the TA‐muscle.

## Conflict of Interest

None declared.
